# Effect of Dapagliflozin on Indicators of Myocardial Fibrosis and Levels of Inflammatory Factors in Heart Failure Patients

**DOI:** 10.1155/2022/5834218

**Published:** 2022-09-05

**Authors:** Chuanqiang Wang, Yiteng Qin, Xiaojun Zhang, Yang Yang, Xuan Wu, Jing Liu, Shuhui Qin, Ke Chen, Wenliang Xiao

**Affiliations:** ^1^Department of Cardiology, The Third Hospital of Hebei Medical University, 050051 Shijiazhuang City, Hebei Province, China; ^2^Basic Medicine College, Hebei Medical University, 050017 Shijiazhuang City, Hebei Province, China; ^3^Hebei Orthopedic Research Institution, 050051 Shijiazhuang City, Hebei Province, China; ^4^Catheter Room, Third Hospital of Hebei Medical University, 050051 Shijiazhuang City, Hebei Province, China

## Abstract

**Objective:**

To explore the effect of dapagliflozin on the myocardial fibrosis and the levels of inflammatory factors in heart failure patients.

**Methods:**

60 patients with T2DM who were diagnosed as acute left heart failure or acute exacerbation of chronic left heart failure in the Department of Cardiology of our hospital from November 1, 2020, to December 31, 2021, during hospitalization were the study subjects. According to the treatment regimen, they were divided into the experimental group (EG) which received dapagliflozin and conventional drugs and the control group (CG) which received conventional drugs, with 30 cases in each group to compare and analyze the clinical indicators such as myocardial fibrosis and inflammatory factors.

**Results:**

The levels of TNF-*α*, IL-1*β*, IL-6, and hs-CRP in the two groups were decreased gradually after treatment, and the levels of TNF-*α*, IL-1*β*, IL-6, and hs-CRP in the EG were visibly lower compared with those in the CG at week 4 of treatment (*P* < 0.05). The cardiac function evaluation of patients showed that the levels of LVEF and LVEDD in both groups were gradually improved after treatment, with a significant difference from the fourth week. In other words, compared with the CG, the LVEF level in the EG was obviously higher (*P* < 0.05), the LVEDD level was distinctly lower (*P* < 0.05), and the levels of ST2, BNP, and MCP-1 in the EG were clearly lower at week 4 of treatment (*P* < 0.05) with a statistical significance in difference.

**Conclusion:**

Dapagliflozin has a definite curative effect in heart failure patients with type 2 diabetes mellitus, which can effectively reduce the inflammatory response of patients and inhibit the myocardial fibrosis, and has a potential value in improving cardiac function and promoting prognosis.

## 1. Introduction

In recent years, diabetes mellitus has become a serious public health problem globally, which can cause vascular endothelial injury and lead to the changes of heart construction and function, and is a high risk factor for heart failure and poor prognosis [1, 2]. Therefore, the development of hypoglycemic programs for patients with diabetes mellitus should not only consider the blood glucose control effect but also prevent the heart failure. Dapagliflozin, as a new hypoglycemic agent and also a sodium-glucose cotransporter2 (SGLT2) inhibitor, can reduce reabsorption of filtered glucose and the renal threshold value of glucose by inhibiting SGLT2, thereby increasing the urine glucose excretion [3, 4]. At present, clinical studies have confirmed that SGLT2 inhibitor can inhibit or reduce renal and hepatic fibrosis, and related animal experiments have also confirmed that SGLT2 inhibitor has a good inhibitory effect on myocardial fibrosis in rats [5, 6]. In addition, in multiple large-scale clinical studies, dapagliflozin has shown a better curative effect in reducing the mortality and rehospitalization rate of heart failure [7, 8]. However, the inflammatory response is an important mechanism of myocardial fibrosis, and a variety of inflammatory factors are involved in the process of myocardial fibrosis, but it is unclear whether SGLT2 inhibitor affects the inflammatory response and whether it inhibits the process of myocardial fibrosis through the procedure of inflammatory response. Based on the current clinical study progress, this study further explores the effect of dapagliflozin on the myocardial fibrosis and the levels of inflammatory factors in heart failure patients in order to provide more directions for the treatment of heart failure. The report is discussed in the succeeding sections.

## 2. Materials and Methods

### 2.1. Inclusion Criteria

The criteria were as follows. (1) All patients met the diagnostic criteria of type 2 diabetes mellitus (T2DM) in the Guideline for the Prevention and Treatment of Type 2 Diabetes Mellitus in China (2017 edition) [9] and the diagnostic criteria of heart failure in the Chinese Guidelines for the Diagnosis and Treatment of Heart Failure in 2018 [10]. (2) The age of patients exceeded 18 years old. (3) The cardiac function was graded as II-IV by the New York Heart Association (NYHA) (the clinical features in grade II showed the slight limitation of physical activity and palpitation, short breath, fatigue, and dyspnea caused by common activities; the clinical features in grade III showed the obvious limitation of physical activity and breathlessness and palpitation caused by slight activity with the sign of mild congestion in organs; and the clinical features in grade IV showed the severe limitation of activities and the occurrence of breathlessness and palpitation at rest with the sign of severe-degree congestion in organs). (4) Patients had complete clinical data and high treatment coordination degree. (5) All patients signed the informed consent. (6) Patients could receive the long-term follow-up visit.

### 2.2. Exclusion Criteria

The criteria were as follows: (1) patients with the diseases such as severe sepsis and acute cerebrovascular disease that could cause cardiac dysfunction; (2) female patients in pregnancy or lactation period; (3) patients with other heart diseases like severe congenital heart disease, myocarditis, and valvular heart disease; (4) patients with malignant tumor, thyroid hyperfunction, or multiple organ failure; (5) patients with acute complication of diabetes mellitus; and (6) patients with psychic cognitive impairment or language communication disorder.

### 2.3. Screening and Grouping of Patients

60 patients with T2DM who were diagnosed as acute left heart failure or acute exacerbation of chronic left heart failure in the Department of Cardiology of our hospital from November 1, 2020, to December 31, 2021, during hospitalization were the study subjects, and they were divided into the experimental group (EG) and the control group (CG) according to the treatment regimen, with 30 cases in each group. This study was in line with the criteria of ethics and morality and was adopted after discussion by the ethics committee of our hospital.

### 2.4. Methods

#### 2.4.1. Medication Regimen

Patients in the CG were treated with conventional drug treatment, that is to say, giving them antidiabetic drugs such as metformin and insulin according to the individualized situation of patients. At the same time, beta-adrenergic blockade, calcium channel blockers, diuretics, and digitalis glycosides were used for antiheart failure treatment, and the diet guidance with low glucose and low sodium was given.

Patients in the EG were treated with dapagliflozin (specification: 10 mg∗30 s; manufacturer: AstraZeneca Pharmaceuticals Co., Ltd.; NMPA approval no. J20170040) on the basis of conventional medication regimen at a dose of 5 mg in the morning once a day.

#### 2.4.2. Sample Collection

10-15 ml of venous blood was collected before entering the group with the spontaneous coagulation at room temperature for 10-20 minutes and was centrifuged for about 20 minutes (2000-3000 r/min) to carefully collect the clear supernatant and then was sent to experiment center of our hospital for inspection. The specimens were repeatedly collected at week 1 and week 4 of treatment.

### 2.5. Observation Indices

1. The general information includes age, gender, systolic pressure, heart rate, duration of diabetes mellitus, course of heart failure, NYHA grade, previous medication history, and complications
2. The blood was collected at admission time and at week 1 and week 4 of treatment for centrifugation to collect the clear supernatant, and the tumor necrosis factor-*α* (TNF-*α*), interleukin-1*β* (IL-1*β*), interleukin-6 (IL-6), high-sensitivity c-reactive protein (hs-CRP), soluble suppression of tumorigenicity 2 (ST2), and b-type natriuretic peptide (BNP) were detected in the department of laboratory. The level of monocyte chemoattractant protein-1 (MCP-1) in serum was detected using enzyme-linked immunosorbent assay (ELISA method)
3. The ultrasound cardiography (UCG) examination was performed at admission time and week 1 and week 4 of treatment to analyze statistically the left ventricle ejection fraction (LVEF) and left ventricular end diastolic dimension (LVEDD)

### 2.6. Statistical Treatment

The software SPSS 23.0 was used for the data processing in this study to calculate the differences in data between the two groups, and GraphPad Prism 7 (GraphPad Software, San Diego, USA) was used for chart production. The study data including enumeration and measurement were tested by *x*^2^ and *t*-test, indicated by [*n* (%)] and $\left(\bar{x}\pm s\right)$, which were in line with the normal distribution. When statistical results were *P* < 0.05, the differences were considered to be statistically significant.

## 3. Results

### 3.1. General Information

The age, gender, systolic pressure, heart rate, duration of diabetes mellitus, course of heart failure, NYHA grade, previous medication history, complications, and other general information in both groups were counted, with no statistical significance between the two groups (*P* > 0.05). See details in Table 1.

### 3.2. Inflammatory Factors

According to the statistical data of inflammatory factors in Table 2, the levels of TNF-*α*, IL-1*β*, IL-6, and hs-CRP in the two groups were decreased gradually after treatment, and there were slight differences in the levels of inflammatory factors between the two groups at week 1 of treatment, with no significant difference (*P* < 0.05), while the levels of TNF-*α*, IL-1*β*, IL-6, and hs-CRP in the EG were significantly lower compared with the CG at week 4 of treatment (*P* < 0.05).

### 3.3. Cardiac Function

**Figure 1:**
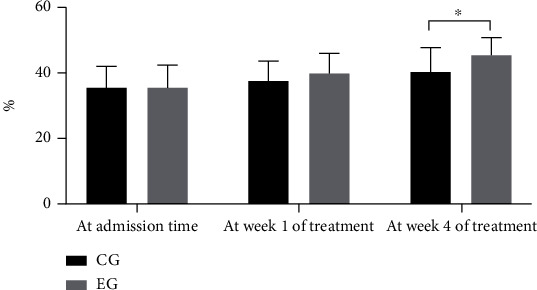
LVEF level of patients. Notes: the horizontal coordinate represented time points, and the horizontal coordinate represented LVEF level (%). The levels of LVEF at admission time and at week 1 and week 4 of treatment in the CG were 35.49 ± 6.55, 37.60 ± 5.91, and 40.25 ± 7.43, respectively. The levels of LVEF at admission time and at week 1 and week 4 of treatment in the EG were 35.57 ± 6.78, 39.85 ± 6.04, and 45.36 ± 5.46, respectively. ^∗^An apparent difference in the levels of LVEF between the two groups at week 4 of treatment (*t* = 3.035, *P* = 0.004).

**Figure 2:**
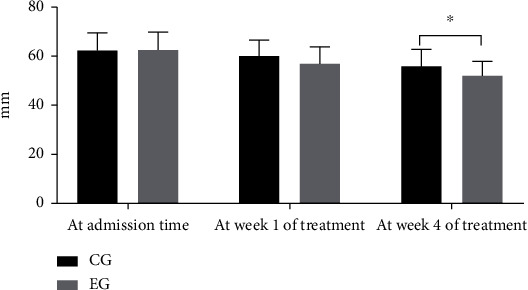
LVEDD level of patients. Notes: the horizontal coordinate represented time points, and the horizontal coordinate represented LVEDD level (mm). The levels of LVEDD at admission time and at week 1 and week 4 of treatment in the CG were 62.40 ± 7.15, 60.11 ± 6.54, and 55.88 ± 7.01, respectively. The levels of LVEDD in the EG at admission time and at week 1 and week 4 of treatment were 62.55 ± 7.24, 56.84 ± 7.07, and 52.10 ± 5.73, respectively. ^∗^An apparent difference in the levels of LVEDD between the two groups at week 4 of treatment (*t* = 2.287, *P* = 0.026).

The cardiac function evaluation of patients showed that the levels of LVEF and LVEDD in both groups were gradually improved after treatment with a significant difference from the fourth week. In other words, compared with the CG, the LVEF level in the EG was obviously higher (*P* < 0.05), and the LVEDD level was distinctly lower (*P* < 0.05). See details in Figures 1 and 2.

### 3.4. Myocardial Fibrosis

At week 4 of treatment, the levels of ST2, BNP, and MCP-1 in the EG were obviously lower than those in the CG (*P* < 0.05) with a statistical significance in difference. See details in Table 3.

## 4. Discussion

Heart failure is a serious and terminal stage of various heart diseases, which is one of the most important cardiovascular diseases today with high morbidity and mortality despite the continuous development of medical technology. The two key processes in the progression of heart failure caused by the reconstruction of myocardial pathology are one of the main pathogenesis mechanism of heart failure, that is, the occurrence of myocardial death (necrosis, apoptosis, and autophagy), such as acute myocardial infarction and severe myocarditis on the one hand, and, on the other hand, system response caused by excessive activation of neuroendocrine system, in which myocardial fibrosis is an important pathological change in the process of heart failure [11, 12]. Therefore, cutting off or delaying the two key processes is an important means to effectively prevent and treat heart failure. SGLT2 inhibitor is a new type of hypoglycemic agent, and various SGLT2 inhibitors can significantly reduce the left ventricle ejection fraction of patients in several large-scale recently finished and underway clinical studies, which is expected to reduce the mortality of heart failure patients. More importantly, these benefits can be observed in patients with or without diabetes mellitus, suggesting that the benefits are independent of the effects on reducing blood glucose [13, 14]. The tablet of oral dapagliflozin (a SGLT2 inhibitor) has been approved by the Food and Drug Administration (FDA) used for the treatment of heart failure on May 5, 2020 (CAS registration no. 461432-26-8), mainly for the heart failure patients with reduced ejection fraction, which is aimed at reducing the risk of cardiovascular mortality and admission due to heart failure of patients. At present, dapagliflozin has been marketed in China, but it is only approved for the treatment of adults with type 2 diabetes mellitus, and there are few related study reports in China. However, our province, as an area with high incidence of cardiovascular disease, has many heart failure patients, and it is worth further exploring the inhibitory effect of dapagliflozin on myocardial fibrosis in heart failure patients, so as to provide more scientific and accurate guidance for the treatment and prognosis of heart failure.

More and more studies have shown that immune inflammatory response exerts a vital role in the process of myocardial fibrosis so that it may be a new way to improve the myocardial fibrosis by regulating the abnormal immune inflammatory response [15]. Inflammation is an important factor inducing the myocardial fibrosis, and inflammatory response and myocardial fibrosis often coexist in the same lesion sites in a variety of cardiovascular diseases. When the damage occurs in the heart, the immune system activation releases a variety of inflammatory factors and activates cardiac fibroblasts leading to the abnormal collagen metabolism and myocardial necrosis and degeneration, thus causing the pathological changes such as myocardial fibrosis. Therefore, the short-term therapeutic target of heart failure alleviates the condition by improving the clinical symptoms of patients, while the long-term therapeutic target improves the survival prognosis of patients with heart failure by reversing the target organ damage such as myocardial fibrosis caused by multiple adverse factors like immune inflammatory response [16, 17]. In this study, the levels of TNF-*α*, IL-1*β*, IL-6, and hs-CRP in the two groups were decreased gradually after treatment, and the levels of TNF-*α*, IL-1*β*, IL-6, and hs-CRP in the EG were obviously lower than those in the CG at week 4 of treatment (*P* < 0.05). The inflammatory cascade reaction mediated by neutrophil granulocyte and mononuclear macrophages is one of the common mechanisms leading to the myocardial fibrosis, which can secrete a variety of proinflammatory cytokines such as TNF-*α*, IL-1*β*, and IL-6, leading to the myocardial fibrosis. The results of this study showed that dapagliflozin can effectively inhibit the inflammatory response in heart failure patients in the short term and reduce the levels of inflammatory factors, thus alleviating myocardial fibrosis. The clinical studies have found that the clinical pharmacological mechanism of dapagliflozin in the treatment of heart failure includes the following points. (1) Dapagliflozin can effectively reduce ventricular hypertrophy and restore dilation function. (2) The apoptosis of cardiomyocytes was inhibited, thus delaying the progress of diastolic dysfunction, and the activation of mitochondrial autophagy is an important mechanism for mediating the protective mechanism produced by dapagliflozin. (3) Dapagliflozin also reduces the oxidative stress reaction through Nrf2/ARE and other pathways, so as to hinder myocardial fibrosis and ventricular hypertrophy, improve myocardial dilation function, and promote the myocardial remodeling and improvement effect of myocardial microcirculation. The cardiac function evaluation of patients showed that the levels of LVEF and LVEDD in the two groups were gradually improved after treatment, with a significant difference from the fourth week. In other words, compared with the CG, the LVEF level in the EG was obviously higher (*P* < 0.05), and the LVEDD level was distinctly lower (*P* < 0.05), which was consistent with the results of previous studies [18, 19], confirming that dapagliflozin is helpful to improve the cardiac function of heart failure patients. Dapagliflozin can promote the excretion of sugar and sodium in patients, with an effect on osmotic diuresis, thereby reducing the cardiac preload, while it also reduces the cardiac afterload by reducing the blood pressure and arterial stiffness and improving the vascular endothelial function, thereby improving the cardiac diastolic function.

In this study, the levels of ST2, BNP, and MCP-1 in the EG were overtly lower than those in the CG (*P* < 0.05) at week 4 of treatment, indicating that dapagliflozin has an obvious inhibitory effect on myocardial fibrosis in heart failure patients with type 2 diabetes mellitus. Besides reducing the blood glucose, dapagliflozin also has multiple cardiovascular protective effects and alleviates the heart failure symptoms mainly by early hemodynamic changes. The possible mechanisms are as follows. (1) The cardiovascular risk factors were improved, including reducing the BMI, blood lipid, systolic pressure, and blood uric acid level, postponing the kidney disease, and reducing the excretion of urine protein [20–22]. (2) The vasodilation function in patients was improved to increase the amount of blood pump. Previous studies have shown that dapagliflozin can reduce the myocardial oxidative stress and inhibit the expression of inflammatory factors like TNF-*α*, IL-1*β*, IL-6, and MCP-1 in myocardium by the reduction of malonaldehyde and the increase of superoxide dismutase, thus inhibiting the myocardial hypertrophy and myocardial fibrosis [23–25]. (3) Dapagliflozin enables to improve the hemodynamics through osmotic diuresis and improving the arteriosclerosis, thus reducing the cardiac preload and afterload.

The shortcomings of this study were as follows. (1) Dapagliflozin may benefit heart failure patients through multiple mechanisms, but SGLT2 is not expressed in human cardiomyocytes, mainly distributed in the kidney, and it is not clear whether dapagliflozin directly affects the cardiomyocytes, which needs to be supported by a large number of experimental results. (2) In this study, the sample size is small and the long-term efficacy of patients is not tracked, and large sample studies are still needed to further clarify its clinical value. (3) At the same time, limited by the research conditions, this study lacked the study on the adverse reactions of patients after medication, so this problem should be paid attention to in the follow-up studies.

In summary, dapagliflozin has a definite curative effect on heart failure patients with type 2 diabetes mellitus, which effectively reduces the levels of inflammatory factors in patients and improves the cardiac function, and further studies will help to establish a better solution for such patients.

## Figures and Tables

**Table 1:** Comparison of general information in patients between the two groups (*n* = 30).

Observation indices	CG	EG	*x* ^2^/*t*	*P*
Age (years)	67.50 ± 6.90	67.43 ± 6.70	0.040	0.968
Gender (male/female)	17/13	18/12	0.069	0.793
Systolic pressure (mmHg)	125.63 ± 8.67	127.00 ± 9.18	0.594	0.555
Heart rate (times/min)	68.53 ± 4.07	68.87 ± 4.20	0.318	0.751
Duration of diabetes mellitus (years)	9.41 ± 3.16	9.24 ± 3.24	0.206	0.834
Course of heart failure (months)	8.17 ± 2.73	8.73 ± 2.58	0.817	0.418
NYHA grade				
Grade II	10 (33.33)	7 (23.33)	0.739	0.390
Grade III	18 (60.00)	20 (66.67)	0.287	0.592
Grade IV	2 (6.67)	3 (10.00)	0.218	0.640
Previous medication history				
Dipeptidyl peptidase-4 inhibitor	22 (73.33)	23 (76.67)	0.089	0.766
Sulfonylurea	9 (30.00)	8 (26.67)	0.082	0.774
Alpha glucosidase inhibitor	7 (23.33)	8 (26.67)	0.089	0.766
Nitrotyrosine	10 (33.33)	12 (40.00)	0.287	0.592
Biguanides	15 (50.00)	13 (43.33)	0.268	0.605
ACEI/ARB	24 (80.00)	25 (83.33)	0.111	0.739
Calcium channel blockers	8 (26.67)	10 (33.33)	0.318	0.573
Beta-adrenergic blockade	15 (50.00)	14 (46.67)	0.067	0.796
Atorvastatin	22 (73.33)	19 (63.33)	0.693	0.405
Complications				
Hypertension	9 (30.00)	10 (33.33)	0.077	0.781
COPD	4 (13.33)	5 (16.67)	0.131	0.718
Hyperlipidemia	7 (23.33)	5 (16.67)	0.417	0.519

**Table 2:** Levels of inflammatory factors in patients.

Inflammatory factors		CG	EG	*t*	*P*
TNF-*α* (ng/l)	At admission time	49.26 ± 7.66	48.82 ± 7.35	0.227	0.821
	At week 1 of treatment	43.11 ± 7.15	40.72 ± 7.03	1.306	0.197
	At week 4 of treatment	35.33 ± 8.17	28.10 ± 6.30	3.838	<0.001
IL-1*β* (ng/ml)	At admission time	745.2 ± 84.1	743.8 ± 84.8	0.064	0.949
	At week 1 of treatment	572.7 ± 72.8	531.9 ± 78.5	1.883	0.065
	At week 4 of treatment	497.5 ± 74.3	356.4 ± 60.2	8.082	<0.001
IL-6 (ng/l)	At admission time	150.9 ± 15.4	151.3 ± 15.7	0.100	0.921
	At week 1 of treatment	138.9 ± 12.3	132.4 ± 13.8	1.926	0.059
	At week 4 of treatment	120.5 ± 11.2	107.8 ± 9.5	4.736	<0.001
hs-CRP (mg/L)	At admission time	9.88 ± 2.28	9.64 ± 2.51	1.908	0.061
	At week 1 of treatment	9.10 ± 2.13	8.86 ± 2.01	0.449	0.655
	At week 4 of treatment	7.59 ± 1.01	6.87 ± 1.05	2.707	0.009

**Table 3:** Indicators of myocardial fibrosis in patients.

Inflammatory factors		CG	EG	*t*	*P*
ST2 (*μ*g/l)	At admission time	52.25 ± 4.62	51.82 ± 4.53	0.821	0.415
	At week 1 of treatment	49.21 ± 4.68	47.10 ± 4.17	1.844	0.070
	At week 4 of treatment	45.82 ± 3.15	39.15 ± 3.02	8.372	<0.001
BNP (pmol/l)	At admission time	75.59 ± 9.52	74.90 ± 9.83	0.276	0.783
	At week 1 of treatment	70.87 ± 9.14	66.29 ± 9.20	1.934	0.058
	At week 4 of treatment	59.12 ± 8.56	49.53 ± 8.25	4.418	<0.001
MCP-1 (ng/l)	At admission time	18.03 ± 4.04	17.95 ± 4.10	0.076	0.940
	At week 1 of treatment	16.55 ± 3.43	15.08 ± 3.30	1.692	0.096
	At week 4 of treatment	13.41 ± 3.21	10.28 ± 1.43	4.878	<0.001

## Data Availability

The data to support the findings of this study is available on reasonable request from the corresponding author.
